# Enhancing the catalytic stability of lead dioxide electrodes for oxygen evolution reaction through α-PbO_2_ lining and β-PbO_2_ coating doped with F and Fe

**DOI:** 10.1039/d6ra05492g

**Published:** 2026-08-10

**Authors:** Mohanna Amini, Milad Rezaei

**Affiliations:** a Department of Materials and Metallurgical Engineering, Amirkabir University of Technology (Tehran Polytechnic) Hafez Ave., P.O. Box 15875-4413 Tehran Iran miladrezaei@aut.ac.ir

## Abstract

This study focuses on the synthesis of a non-noble metal-based anode electrode on a titanium substrate. Lead dioxide anodes were fabricated *via* electrodeposition, employing a two-step process on a tin-antimony oxide (ATO) interlayer deposited by thermal decomposition. The inner α-PbO_2_ layer was electrodeposited from an alkaline solution, while the outer β-PbO_2_ layer was formed from an acidic bath. To augment the catalytic properties and stability of the anode, the effects of fluorine, cobalt, copper, nickel, and iron additives were systematically investigated. Initially, 0.02 M sodium fluoride was introduced into the β-PbO_2_ electrodeposition bath. Subsequently, 0.01 M solutions of cobalt, copper, nickel, and iron nitrates were added into the lead nitrate and sodium fluoride deposition solution. The phase structure, surface electronic states, and morphology of the synthesized materials were thoroughly characterized using X-ray diffraction (XRD), X-ray photoelectron spectroscopy (XPS), and field-emission scanning electron microscopy (FE-SEM) coupled with energy-dispersive X-ray spectroscopy (EDS). Catalytic activity and electron transfer resistance were evaluated by linear sweep voltammetry (LSV) and electrochemical impedance spectroscopy (EIS). Electrode stability was assessed *via* chronopotentiometry (CP) and cyclic voltammetry (CV). While fluorine doping alone diminished catalytic properties, it enhanced the anode's stability. Co-doping with fluorine and iron resulted in reduced grain size and increased specific surface area. The fluorine–iron co-doped lead dioxide anode demonstrated superior catalytic performance, exhibiting a charge transfer resistance of 20 Ω cm^2^ and an operational stability of 66 hours at a current density of 3 A cm^−2^, significantly outperforming the lead dioxide anode.

## Abstract 2

This study focuses on the synthesis of a non-noble metal-based anode electrode on a titanium substrate.

## Graphical abstract

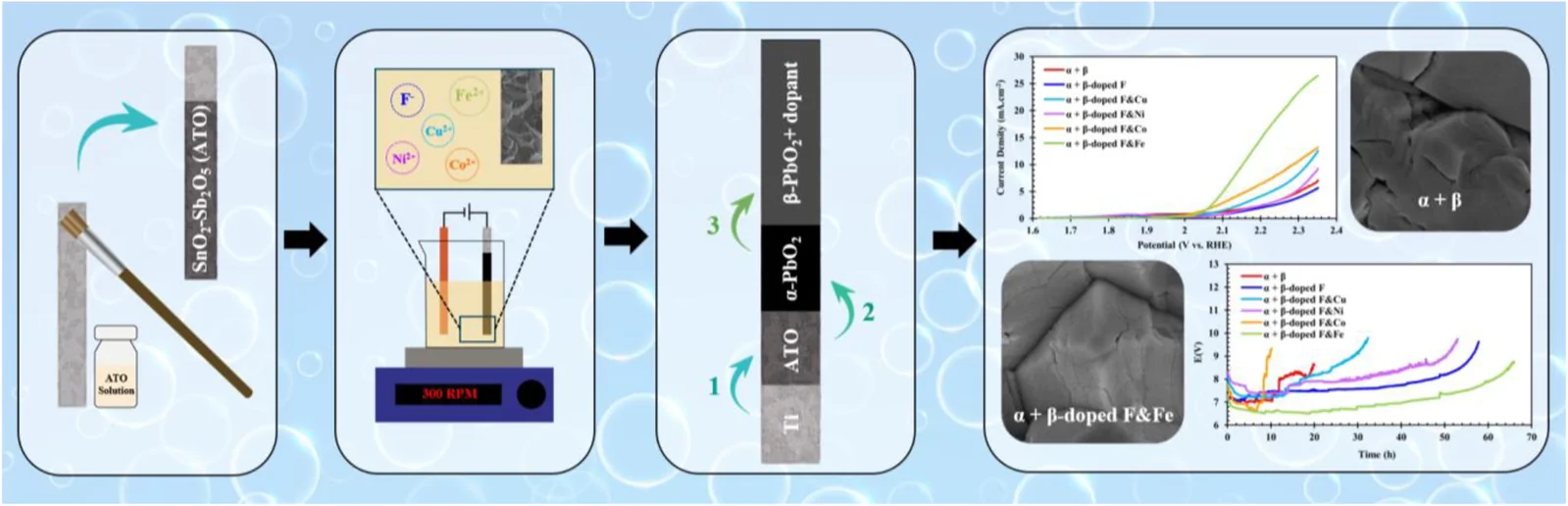

## 1 Introduction

The oxygen evolution reaction (OER) is a critical process in numerous engineering applications, including metal electrowinning,^1^ hydrogen fuel production,^2^ and cathodic protection.^3^ Consequently, the development of OER electrodes that are both catalytically efficient and economically viable is paramount. An effective OER electrode necessitates an electrocatalyst with low overpotential, high activity, cost-effectiveness, and exceptional stability. Ideal electrodes should sustain high-rate anodic reactions and demonstrate long-term durability in acidic electrolytes. While Ru, Ir, and Rh-based catalysts exhibit the highest OER activity,^4,5^ their high cost and scarcity preclude large-scale application,^6^ underscoring the need for cost-effective OER catalysts based on transition metals. Among various candidates, PbO_2_ stands out as a promising anode electrocatalyst.^7–9^ Lead dioxide is an ideal material for anode electrocatalysts due to its low cost, facile synthesis, and remarkable corrosion resistance in acidic media.^10^

Electrodeposited lead dioxide primarily exists in two crystalline forms: orthorhombic α-PbO_2_ and tetragonal β-PbO_2_. The β-PbO_2_ phase, synthesized from acidic solutions, demonstrates superior catalytic activity compared to its α-phase counterpart obtained from alkaline baths.^11^ Depositing β-PbO_2_ onto an α-PbO_2_ interlayer enhances its crystallization due to the fine particle size of the underlying α-PbO_2_.^12^ Multi-component anodes incorporating β-PbO_2_ are employed to improve stability and extend service life.^11^ Common dopants used to modify the structure and performance of β-PbO_2_ include Bi^3+^,^13^ Ni^2+^,^14^ Ag^+^,^15^ Ce^3+^,^16^ and Co^2+^.^17,18^ Substrates for these electrodes typically comprise inert materials such as titanium,^19^ precious metals,^20^ stainless steel,^21^ and graphite.^22^ Titanium (Ti) is an attractive substrate for electrolysis applications due to its cost-effectiveness, reusability, and conductive properties.^23^ Furthermore, applying a SnO_2_–Sb_2_O_5_ (ATO) coating on titanium electrodes enhances chemical stability, electrocatalytic activity, and facilitates the anodic electrodeposition process.^24^

Numerous efforts have been directed toward developing PbO_2_ as a highly stable active electrode. Key parameters influencing electrode properties include the electrolyte composition, temperature, current density/voltage, substrate material, interlayer composition, synthesis method, additives, and electrodeposition time. Despite recent advancements, fabricating a cost-effective anode with high durability for OER remains a significant challenge. Moreover, by reviewing the mentioned papers, it is clear that most of them have evaluated the catalytic properties and investigated the effect of dopant on improving the OER activity. Therefore, there is a need for a research work that evaluates and targets the life and stability of the electrocatalyst for the OER in an acidic medium by development a lead dioxide lining and then adding a dopant inside the lead dioxide overlaying. Therefore, this study aims to develop an efficient, active, and highly stable lead dioxide-based electrode for the OER in acidic environments. In the first phase of this work, the baseline effect of the underlying α-PbO_2_ lining on both the electrocatalytic performance and long-term operational stability of the electrode was systematically evaluated. A strategic two-step electrodeposition pathway was employed to fabricate α-PbO_2_ and β-PbO_2_ layers. F^−^, Cu^2+^, Ni^2+^, Co^2+^, and Fe^2+^ ions were utilized as dopants to modify β-PbO_2_ electrodes on a Ti/SnO_2_–Sb_2_O_5_/α-PbO_2_ substrate. The influence of these dopants on electrode morphology and structure was investigated using X-ray diffraction (XRD) and field emission scanning electron microscopy (FE-SEM). Furthermore, X-ray photoelectron spectroscopy (XPS) was employed to definitively determine the surface electronic structures, chemical bonding states, and valence configurations of the integrated F^−^ and Fe^3+^ dopants within the optimized multi-component oxide lattice. Electrochemical performance, including the impact of varied dopant configurations on the performance enhancement of the PbO_2_ electrodes, was systematically evaluated through linear sweep voltammetry (LSV), electrochemical impedance spectroscopy (EIS), chronopotentiometry (CP), and cyclic voltammetry (CV). Subsequently, the electrochemical active surface area (ECSA) was quantitatively determined through scan-rate dependent cyclic voltammetry, while the long-term structural and morphological stability was rigorously examined post-durability testing.

## 2 Material and methods

### 2.1 Materials

All chemicals used were of analytical reagent grade and were employed without further purification. Aqueous solutions were prepared using deionized water. A titanium plate (99.5% purity, 20 mm × 10 mm × 0.5 mm) served as the substrate, while a copper plate of equivalent surface area was used as the cathode.

### 2.2 Methods

#### 2.2.1 Electrode preparation

Titanium plates were initially sandblasted to create a roughened surface, enhancing coating adhesion. The plates subsequently underwent ultrasonic cleaning in a 1 M NaOH solution for 15 minutes, followed by etching in a boiling 10 wt% oxalic acid solution for 60 minutes. The etched plates were thoroughly rinsed with deionized water, dried, and immediately brush-coated with an ATO solution. The ATO solution was prepared from 5 cc *n*-butanol, 5 cc isopropanol, 5 cc ethanol, 1 cc hydrochloric acid, 1 wt% SbCl_3_, and 10 wt% SnCl_4_·6H_2_O. This coating procedure—brushing, drying at 100 °C, and calcining at 450 °C for 10 minutes—was repeated ten times. Finally, the sample was annealed at 450 °C for 60 minutes.

The PbO_2_ electrodeposition was conducted in two sequential steps. First, an α-PbO_2_ layer was deposited from an alkaline solution onto the Ti/SnO_2_–Sb_2_O_5_ substrate. Subsequently, the β-PbO_2_ layer was electrodeposited from an acidic solution. To investigate the effect of co-doping, 0.01 M solutions of copper, nickel, cobalt, and iron nitrate salts were added to the deposition bath alongside 0.02 M NaF. Tables 1 and 2 detail the synthesis conditions and chemical compositions of the electrodeposition solutions, respectively.

**Table 1:** Conditions for electrochemical synthesis of the anodes

	Synthesis conditions	
β-PbO_2_	Temperature	65 °C
	Current density	20 mA cm^−2^
	Time	120 min
	Stirrer speed	300 rpm
α-PbO_2_	Temperature	40 °C
	Current density	10 mA cm^−2^
	Time	60 min
	Stirrer speed	300 rpm

**Table 2:** Chemical composition of electrodeposition solution

Sample	Electrodeposition solution
α	3.5 M NaOH + 0.1 M PbO
β	0.5 M Pb(NO_3_)_2_ + 0.3 M HNO_3_
β-doped F	0.5 M Pb(NO_3_)_2_ + 0.02 M NaF + 0.3 M HNO_3_
β-doped F and Co	0.5 M Pb(NO_3_)_2_ + 0.02 M NaF + 0.01 M Co(NO_3_)_2_ + 0.3 M HNO_3_
β-doped F and Cu	0.5 M Pb(NO_3_)_2_ + 0.02 M NaF + 0.01 M Cu(NO_3_)_2_ + 0.3 M HNO_3_
β-doped F and Ni	0.5 M Pb(NO_3_)_2_ + 0.02 M NaF + 0.01 M Ni(NO_3_)_2_ + 0.3 M HNO_3_
β-doped F and Fe	0.5 M Pb(NO_3_)_2_ + 0.02 M NaF + 0.01 M Fe(NO_3_)_2_ + 0.3 M HNO_3_

#### 2.2.2 Characterization

X-ray diffraction (XRD, GNR, EXPLORER) analysis was performed using a diffractometer with Cu-K_α_ radiation (40 kV, 40 mA) to verify the synthesized phases. Morphology and particle size were characterized using field emission scanning electron microscopy (FE-SEM, MIRA3, TESCAN), coupled with energy-dispersive X-ray spectroscopy (EDS) for elemental quantification. The elemental composition and surface chemical states of the optimal synthesized electrode were investigated *via* X-ray photoelectron spectroscopy (XPS) utilizing a Bestec spectrometer (model EA10) to confirm the successful incorporation and electronic environments of the dopants.

All electrochemical measurements were conducted in a three-electrode cell at ambient temperature. The prepared samples served as the working electrode, a platinum plate as the counter electrode, and an Ag/AgCl electrode as the reference electrode. Linear sweep voltammetry (LSV) was performed in a 0.5 M H_2_SO_4_ solution at a scan rate of 10 mV s^−1^ to determine the oxygen evolution potential. Electrochemical impedance spectroscopy (EIS) measurements were carried out at a potential of 1.93 V (*vs.* Ag/AgCl) in 0.5 M H_2_SO_4_, over a frequency range from 10^5^ Hz to 10^−2^ Hz with a 10 mV AC amplitude. The EIS data were fitted using Zview 3.1 software. The small-amplitude cyclic voltammetry (SACV) measurements for ECSA evaluation were carried out in a 0.5 M H_2_SO_4_ solution within a potential window ±10 mV *vs.* OCP at scan rates of 1, 2, 3, and 4 mV s^−1^. Electrode stability was evaluated *via* an accelerated lifetime test (chronopotentiometry, CP) at a current density of 3 A cm^−2^ in 0.5 M H_2_SO_4_ at room temperature; the test concluded when the cell voltage increased by 1.5 V. Cyclic voltammetry (CV) was performed within a potential window of 1.7–2.2 V (*vs.* Ag/AgCl) in 0.5 M H_2_SO_4_ at a scan rate of 100 mV s^−1^ for 2000 cycles.

## 3 Results and discussion

### 3.1 Microstructure, morphology, and composition

**Fig. 1:**
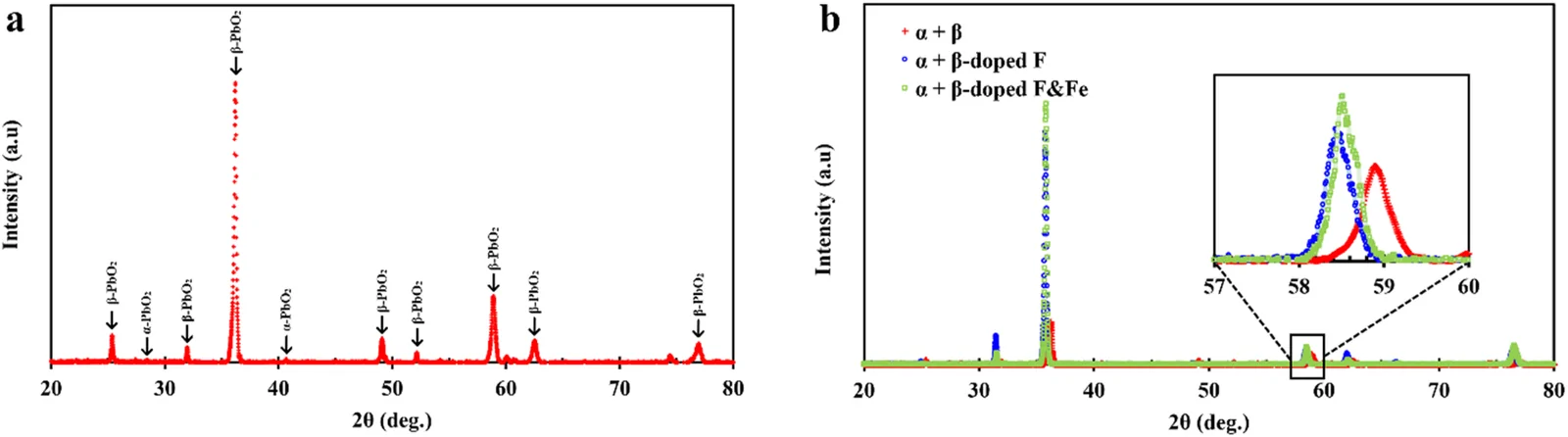
(a) The XRD pattern of α + β sample, α-PbO_2_, and β-PbO_2_ peaks have been identified. (b) The XRD patterns of α + β, α + β-doped F, and α + β-doped F and Fe samples, shifting peaks in samples of α + β-doped F and α + β-doped F and Fe.

Fig. 1a presents the XRD pattern of the pure PbO_2_ electrode, confirming the presence of both α and β phases, as identified in previous studies.^12,25^ The diffraction peaks corresponding to β-PbO_2_ were sharp and well-defined, indicating high crystallinity. In contrast, the α-PbO_2_ peaks were less intense, suggesting smaller grain size and a thinner layer, which can promote the formation of larger β-PbO_2_ crystals.^12^ The β-PbO_2_ phase is preferred for electrocatalytic anodes in high-current applications due to its superior electrical conductivity compared to the α-phase.^26^Fig. 1b displays the XRD patterns for the α + β, F-doped α + β, and F and Fe co-doped α + β electrodes. Notably, the peaks for the F-doped PbO_2_ shifted towards lower angles relative to pure PbO_2_, indicating lattice expansion. The subsequent incorporation of Fe into the F–PbO_2_ matrix induced a slight lattice contraction.^27^

**Fig. 2:**
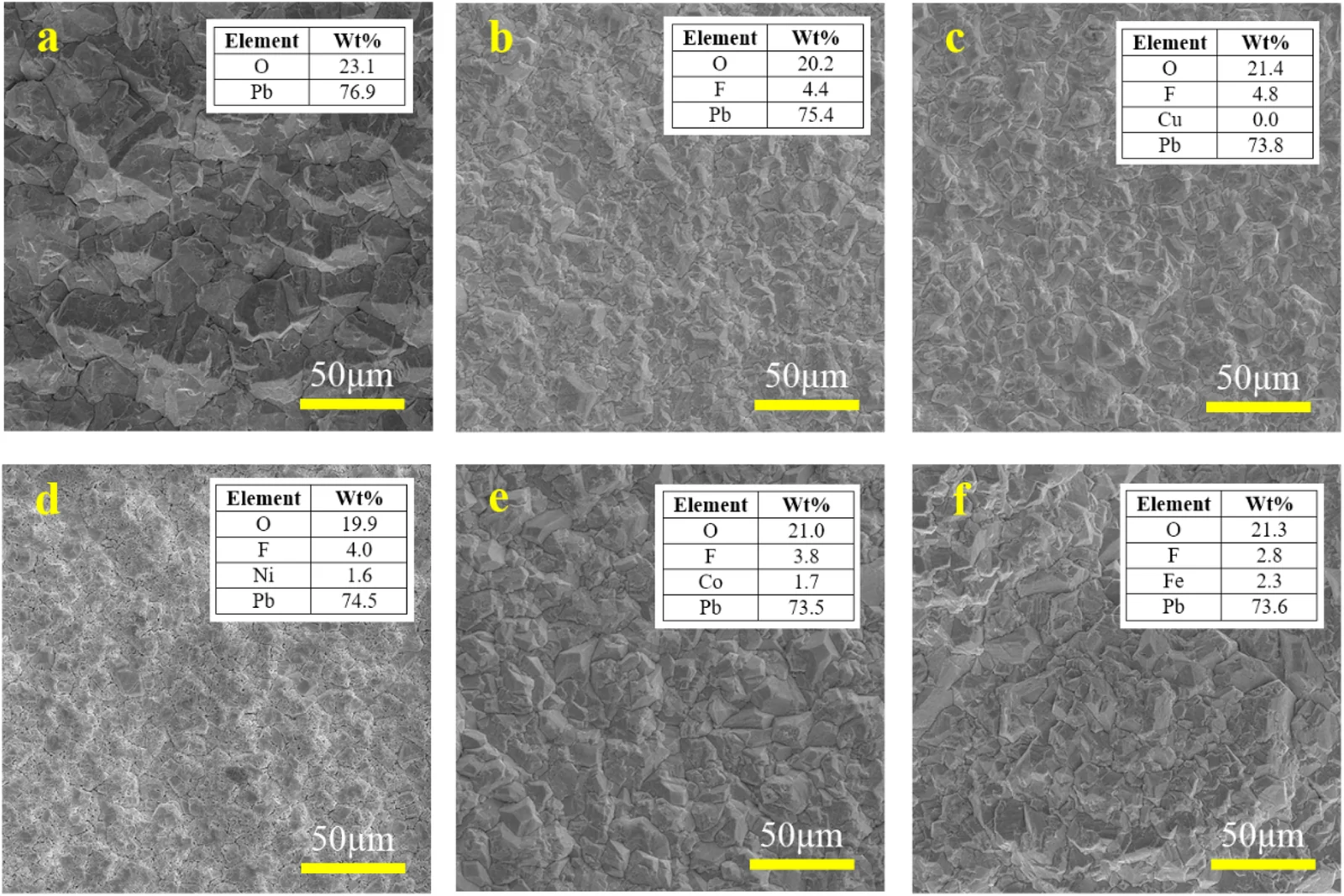
FE-SEM micrographs (scale bar: 50 µm) and corresponding EDS analysis result for the samples, respectively; (a) α + β, (b) α + β-doped F, (c) α + β-doped F and Cu, (d) α + β-doped F and Ni, (e) α + β-doped F and Co, and (f) α + β-doped F and Fe.

Fig. 2 presents FE-SEM micrographs at 50 µm scale, alongside EDS-derived elemental weight percentages. The characteristic pyramidal morphology of PbO_2_ is evident in all samples (a to f). As illustrated in Fig. 2, well-defined pyramidal structures characteristic of lead dioxide (PbO_2_) were successfully formed. Furthermore, the EDS analytical profiles confirm the successful incorporation of fluorine (F) and iron (Fe) heteroatoms, demonstrating their effective integration into the lead dioxide matrix. The high-magnification FE-SEM micrographs (scale bar: 2 µm) are provided in Fig. S1 of the SI.

**Fig. 3:**
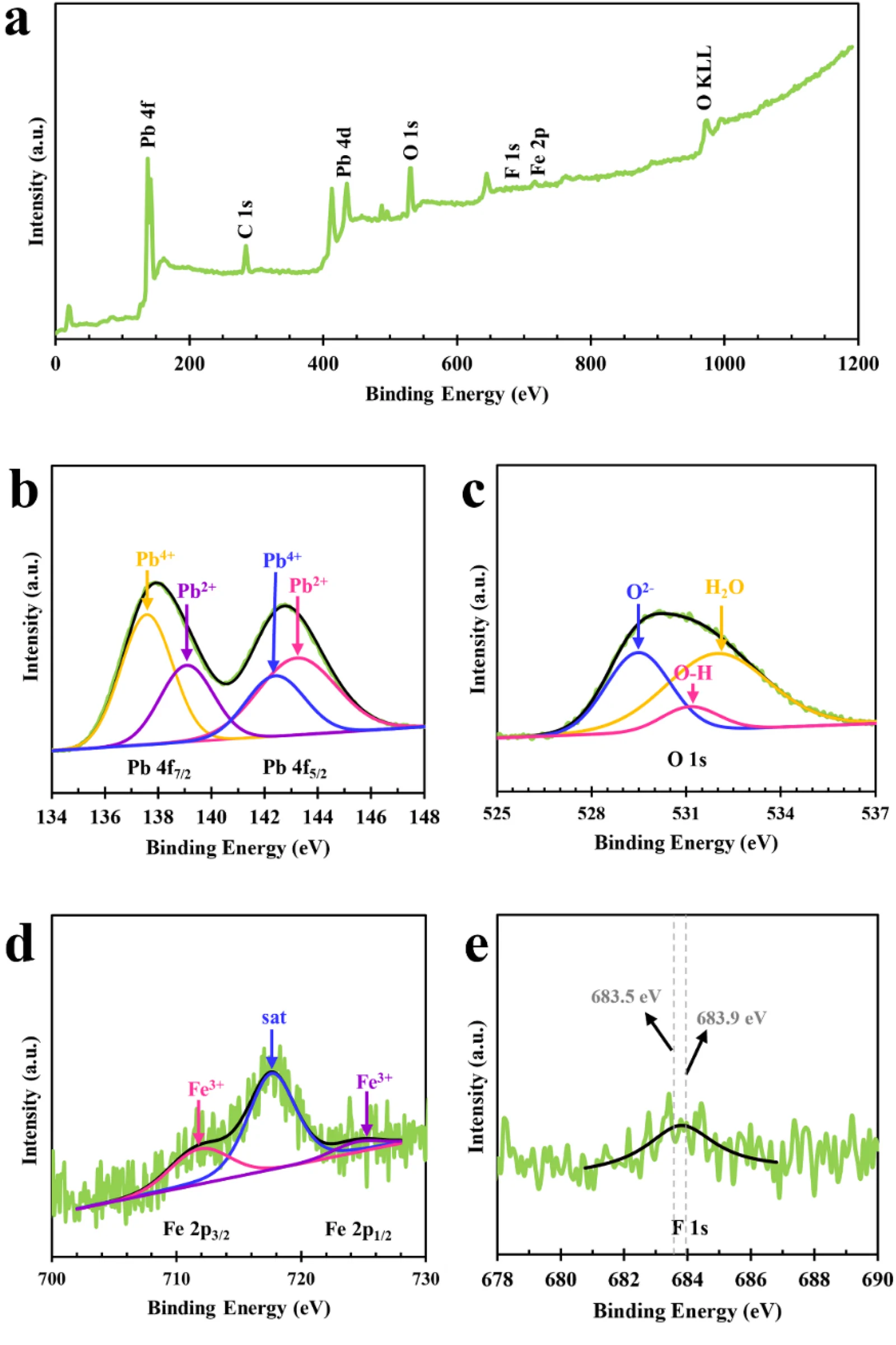
XPS spectra of the optimal α + β-doped F and Fe electrode: (a) wide survey scan, and high-resolution core-level detailed spectra of (b) Pb 4f, (c) O 1s, (d) Fe 2p, and (e) F 1s.

The precise surface composition and chemical valence states of the optimized α + β-doped F and Fe electrode were further investigated using X-ray photoelectron spectroscopy (XPS). The CasaXPS software (version 2.3.25) was utilized for the peak deconvolution and fitting of the core-level spectra. The wide survey spectrum of the electrode (Fig. 3a) confirms that the constituent elements present in the final active coating are exclusively lead (Pb), oxygen (O), iron (Fe), and fluorine (F).^28–31^

In the high-resolution Pb 4f spectrum (Fig. 3b), the primary doublet peaks located at 137.51 eV and 142.34 eV are assigned to the Pb^4+^ state of the PbO_2_ matrix, while the secondary peaks positioned at 139.01 eV and 143.22 eV are attributed to Pb^2+^ species.^32,33^ The coexistence of Pb^4+^ and Pb^2+^ oxidation states implies that during the anodic deposition of the co-doped system, a minor portion of the lead species was not fully converted to stoichiometric PbO_2_. This multi-valent hybrid structure—characterized by the incorporation of Pb(ii) oxides PbO or Pb_3_O_4_ alongside PbO_2_ is highly likely to induce a rich density of structural anionic oxygen vacancies to maintain charge neutrality. Consequently, this electronic configuration is expected to drastically accelerate the oxygen evolution reaction (OER) by acting as shallow electron donors and optimizing intermediate adsorption.

Based on the deconvolution of the O 1s spectrum (Fig. 3c), three distinct oxygen environments were resolved at binding energies of 529.48 eV, 531.12 eV, and 531.98 eV. These peaks are assigned to lattice oxygen (O^2−^), surface hydroxyl species (O–H), and adsorbed/structural water molecules (H_2_O), respectively.^34,35^ The strong contribution of lattice oxygen firmly confirms the successful formation of the active lead dioxide phases.

In the high-resolution Fe 2p spectrum (Fig. 3d), a dominant peak located at 711.85 eV assigned to Fe^3+^ species within the sample. Additionally, a distinct satellite peak associated with Fe 2p_3/2_ is observed at 717.63 eV, which is approximately 5.78 eV higher than the core Fe 2p_3/2_ main peak. The primary contribution of the Fe 2p_1/2_ peak is positioned at around 724.50 eV, which is in excellent agreement with previous literature.^36,37^ These findings demonstrate that Fe^3+^ is the predominant iron species integrated onto the electrode surface. Finally, the F 1s core-level peak is observed at approximately 683.80 eV (Fig. 3e). This binding energy value confirms that fluorine has been successfully incorporated into the lead dioxide lattice structure *via* the replacement of lattice oxygen sites. This binding energy is in excellent agreement with values reported in earlier literature for fluorine doping in metal oxide systems, further validating its successful interstitial or substitutional integration.^31,38^

### 3.2 Catalytic properties of electrodes

To examine the effect of the α-PbO_2_ underlayer, a single-layer β-PbO_2_ electrode was synthesized directly onto the Ti/ATO substrate, and its performance was compared with the bilayer α + β electrode. The electrochemical evaluations, including LSV curves, EIS spectra, and accelerated stability test, are presented in Fig. S2 of the SI. Additionally, the LSV curve of the Ti/ATO substrate is included in Fig. S2b to provide a direct comparison between the bare substrate and the active deposited electrocatalyst. The LSV and EIS data show that both electrodes have very similar electrocatalytic activity. Specifically, the charge transfer resistance (*R*_ct_) was 112 Ω cm^2^ for the single β-PbO_2_ electrode and 108 Ω cm^2^ for the bilayer α + β electrode. However, accelerated stability test at a high current density of 3 A cm^2^ reveal that adding the α-PbO_2_ lining significantly extended the electrode service life from less than 15 hours to 20 hours.

**Fig. 4:**
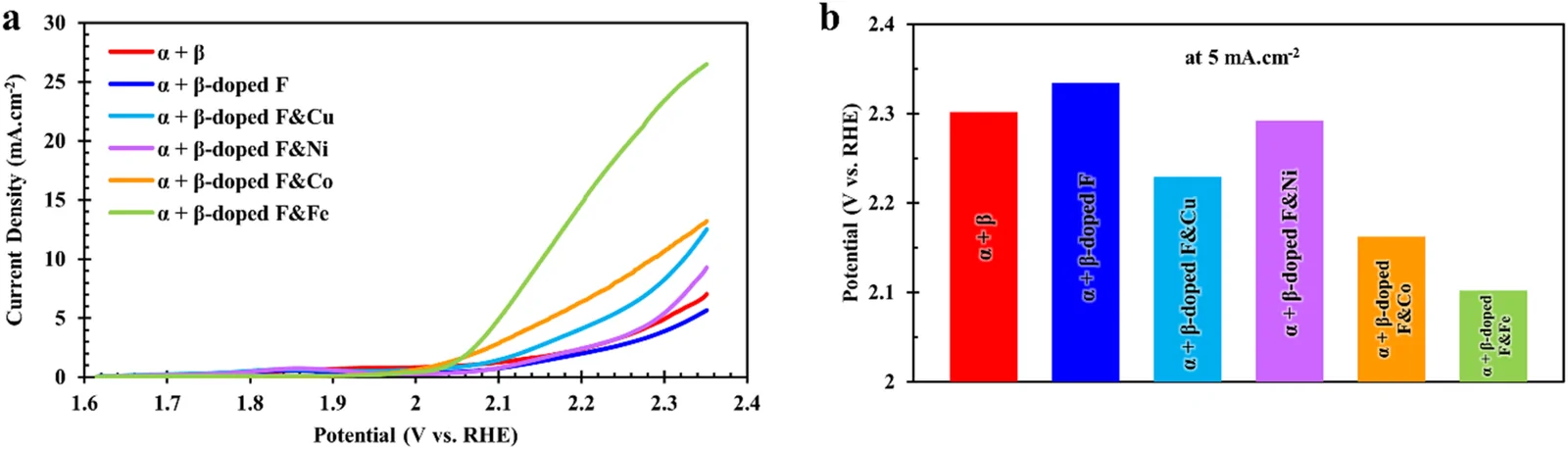
(a) LSV curves of samples in 0.5 M H_2_SO_4_ solution, (b) potential proportional at 5 mA cm^−2^ for all samples.

**Fig. 5:**
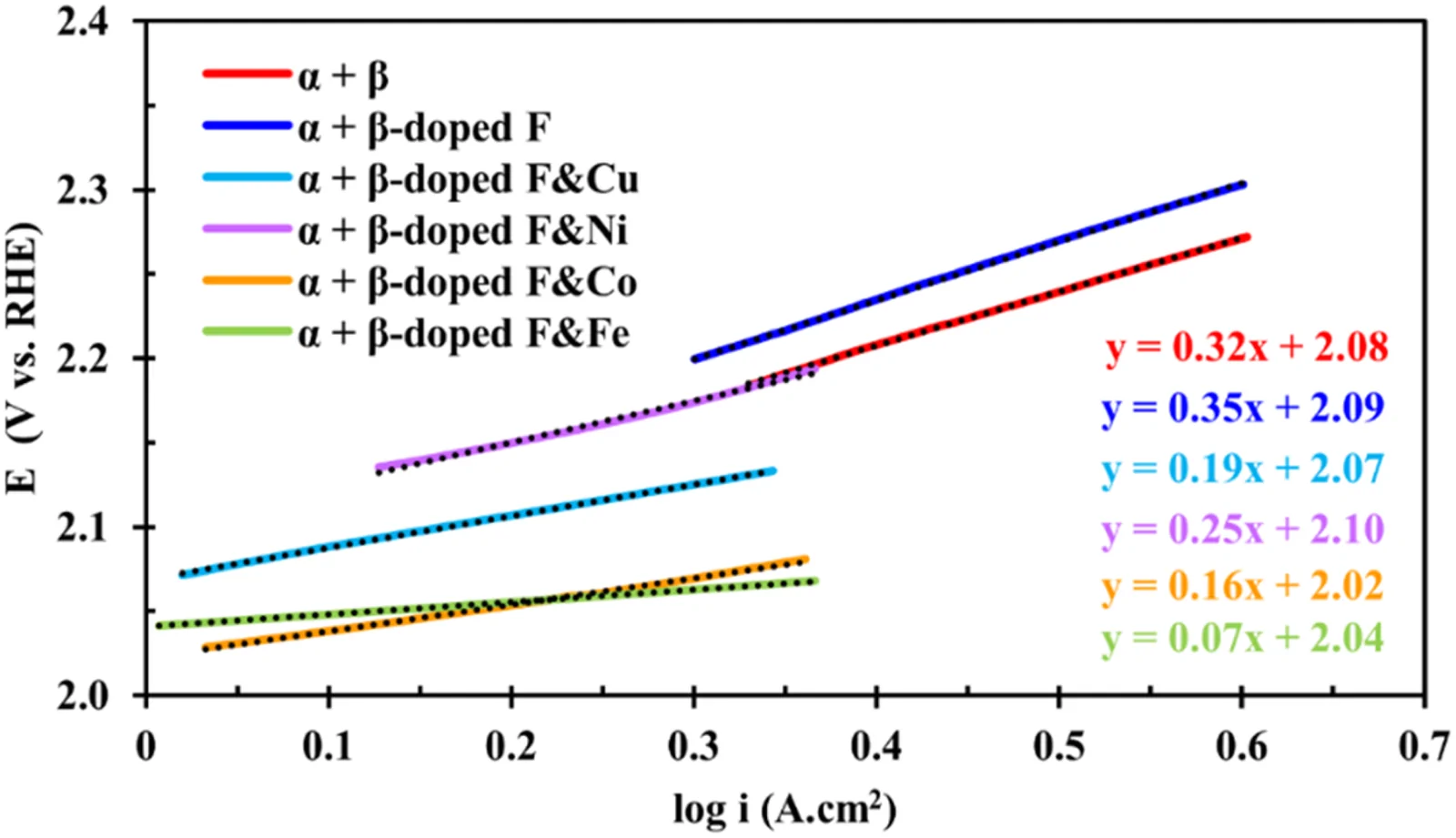
The linear region of E-logi extracted from polarization curves of Fig. 3 present the anodic Tafel constants for all electrodes.

The electrocatalytic activity was assessed using LSV. Fig. 4a shows the LSV curves for all samples tested in 0.5 M H_2_SO_4_ at a scan rate of 10 mV s^−1^. The bar chart in Fig. 4b compares the potentials required to achieve a current density of 5 mA cm^−2^. The F and Fe co-doped sample (α + β-doped F and Fe) required the lowest potential, indicating superior catalytic activity with an overpotential of 2.102 V *vs.* RHE. The anodic Tafel slopes were determined for all samples and plotted in Fig. 5. The lowest Tafel slope was associated with the α + β-doped F and Fe electrode, confirming its enhanced electrocatalytic kinetics.

**Fig. 6:**
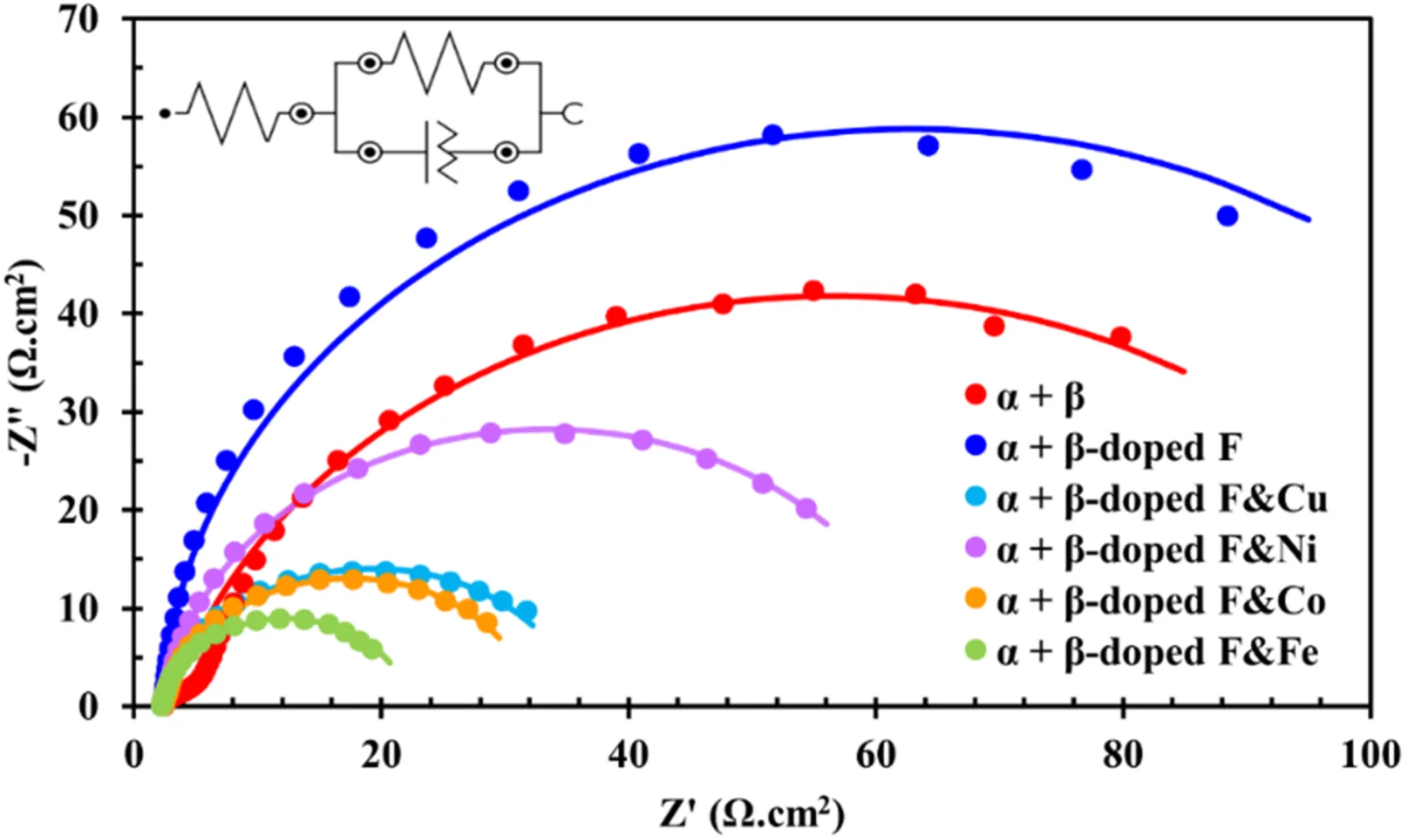
Nyquist diagrams of all electrodes at constant anodic potential of 1.93 V and equivalent circuit.

EIS was employed to investigate the electrochemical properties of the doped and un-doped lead dioxide anodes. Fig. 6 displays the Nyquist plots and the equivalent circuit used for fitting. Qualitatively, the α + β-doped F and Fe electrode exhibits the smallest arc diameter, suggesting lower impedance and facilitated electron transfer due to the synergistic effect of fluorine and iron additives. Table 3 summarizes the fitted parameters, including *R*_s_, *Y*_CPE_, *n*, and *R*_ct_. The charge transfer resistance (*R*_ct_) for the α + β-doped F and Fe electrode was 20 Ω cm^2^, significantly lower than that of other electrodes, indicating superior charge transfer capability and higher activity. This improvement can be attributed to the reduced particle size and increased specific surface area, which provide more active sites.

**Table 3:** EIS parameters obtained from fitting the equivalent circuit

Sample	*R*_s_ (Ω cm^2^)	*Y*_CPE_ (mΩ^−1^ cm^2^ s^*n*^)	*n*	*R*_ct_ (Ω cm^2^)
α + β	2.87	1.21	0.84	108
α + β-doped F	2.36	0.43	0.98	121
α + β-doped F and Co	2.34	0.89	0.88	34
α + β-doped F and Cu	2.47	1.11	0.94	62
α + β-doped F and Ni	2.44	0.58	0.91	30
α + β-doped F and Fe	2.28	0.93	0.93	20

Both the Nyquist plots (Fig. 6) and LSV curves (Fig. 4a) demonstrate that doping with fluorine alone reduces the catalytic properties of the β-PbO_2_ electrode. However, co-doping fluorine with transition metal cations (Ni, Co, Cu, Fe) enhances catalytic performance, with the F and Fe combination yielding the most promising results for developing an active PbO_2_ electrode in acidic media.

To evaluate the electrochemical active surface area (ECSA) of the fabricated anodes without inducing background faradaic currents, small-amplitude cyclic voltammetry (SACV) measurements were systematically conducted. The SACV sweeps were performed at multiple scan rates within a narrow potential window of ±10 mV directly around the open-circuit potential (OCP). By maintaining a small potential amplitude, faradaic oxygen evolution reactions are entirely suppressed, ensuring that the measured current response originates purely from the charging and discharging of the electrochemical double layer. The resulting charging current density (*i*_max_) was plotted against the voltammetric scan rate, where the slope of the linear fit directly determines the double-layer capacitance (*C*_dl_). This obtained *C*_dl_ value was subsequently used to estimate the exact ECSA and evaluate the structural surface area enhancements achieved *via* the co-doping process. The ECSA of a catalyst sample is calculated from *C*_dl_ according to the following equation:^39,40^
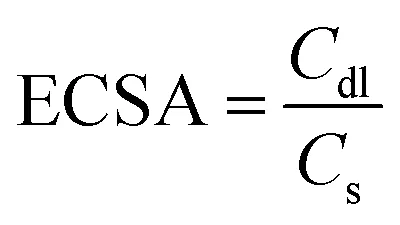


Since the specific capacitance (*C*_s_) of a flat PbO_2_ electrode is widely reported to be around 50 µF cm^−2^ in acidic media, this value was adopted to estimate the ECSA.^40^ The plot of *i*_max_ as a function of the voltammetric scan rate is presented in Fig. S3, and the calculated ECSA values are summarized in Table 4.

**Table 4:** Electrochemical active surface area (ECSA) values for different electrodes

	α + β	α + β-doped F & Fe
ECSA (cm^2^ cm_geo_^−2^)	8.92	12.02

### 3.3 Stability of electrodes

**Fig. 7:**
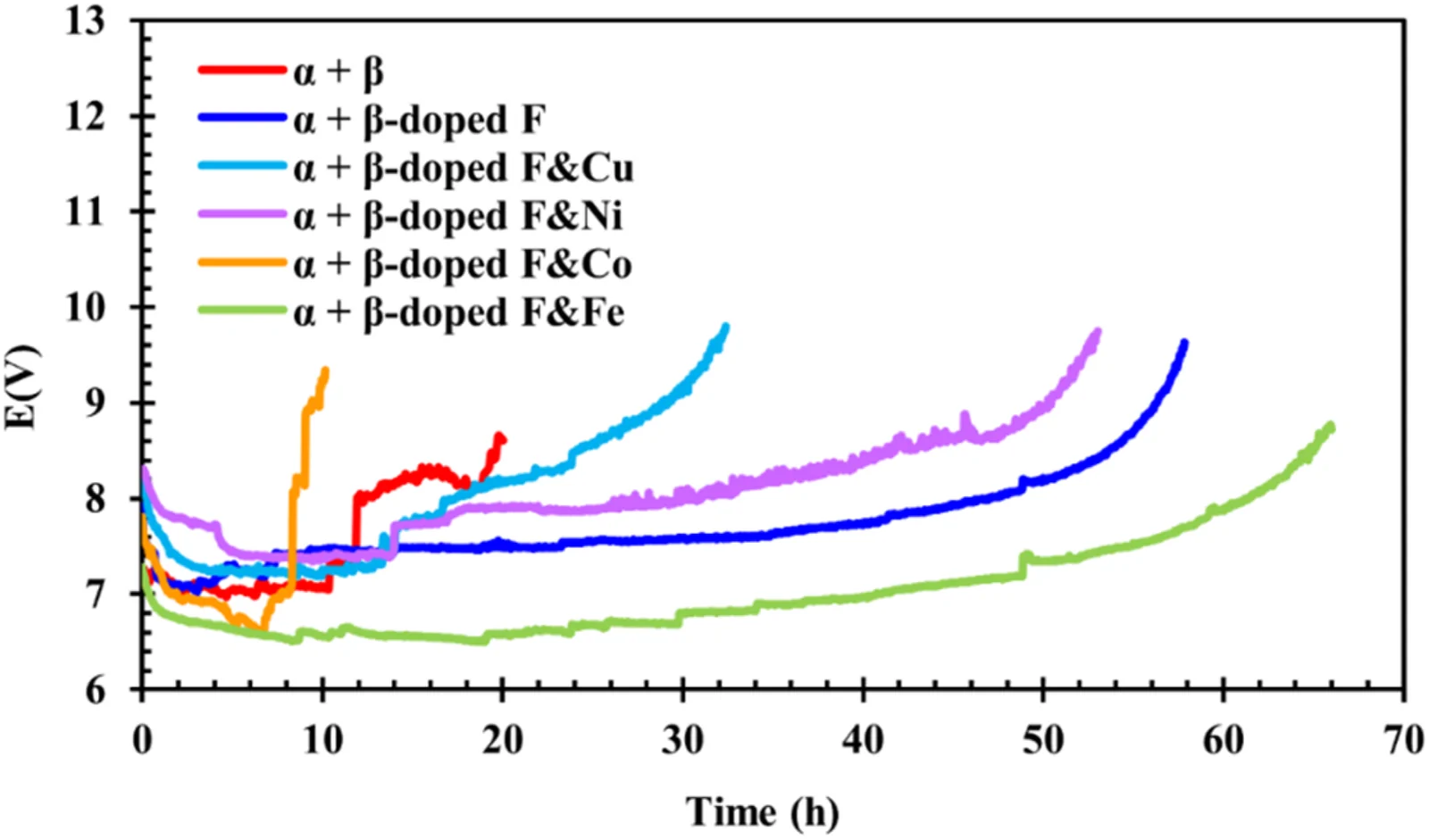
Accelerated life test of all electrodes with an intensified constant current density of 3 A cm^−2^ in 0.5 M H_2_SO_4_.

Electrode stability is a critical performance metric, particularly from an industrial perspective. Accelerated lifetime tests were conducted using chronopotentiometry (CP) at 3 A cm^−2^ in 0.5 M H_2_SO_4_, with failure defined by a 1.5 V increase in cell voltage. The stability curves in Fig. 7 show that the F and Co co-doped sample had the shortest service life. The pure PbO_2_ electrode (α + β) lasted 20 hours, while the F-doped electrode (α + β-doped F) endured for 58 hours. Remarkably, the F and Fe co-doped electrode (α + β-doped F and Fe) exhibited a lifespan of 66 hours under these harsh conditions, which is 3.3 times longer than the pure PbO_2_ electrode. The practical service life under typical operational currents (*e.g.*, 100 mA cm^−2^) would be substantially longer, as service life increases significantly with decreasing current density.^41^ Enhanced stability can also be linked to the alleviation of internal stress within the PbO_2_ matrix.^25^ EDS analysis confirmed the incorporation of F^−^ and Fe^2+^ into the PbO_2_ structure, which likely mitigates internal stress and, combined with grain refinement (Fig. 2), contributes to the extended service life.

Table 5 compares the stability of the lead dioxide electrodes developed in this work with those reported in the literature. The F and Fe co-doped electrode, with a stability value of 198 A cm^−2^ h, demonstrates superior performance compared to other PbO_2_-based anodes listed.

**Table 5:** Comparison of the stability of PbO_2_-based electrodes in some last researches

Sample	Solution	Current density (A cm^−2^)	Stability (A cm^−2^ h)	Reference
FeCN–PbO_2_	3 M H_2_SO_4_	0.5	144	42
PbO_2_-SDS (5)	3 M H_2_SO_4_	0.5	174	10
Fe-doped PbO_2_	3 M H_2_SO_4_	1	116	15
Bi-PbO_2_	1 M H_2_SO_4_	1	156	43
MnO_2_-WC-PbO_2_ (10)	1 M H_2_SO_4_	1	79	44
PbO_2_/SnO_2_-CTAB	2 M H_2_SO_4_	1	72.5	45
Ti/PbO_2_–Tb	0.5 M H_2_SO_4_	0.25	46	46
α + β-doped F and Fe	**0.5 M H**_**2**_ **SO**_**4**_	**3**	**198**	**This study**

**Fig. 8:**
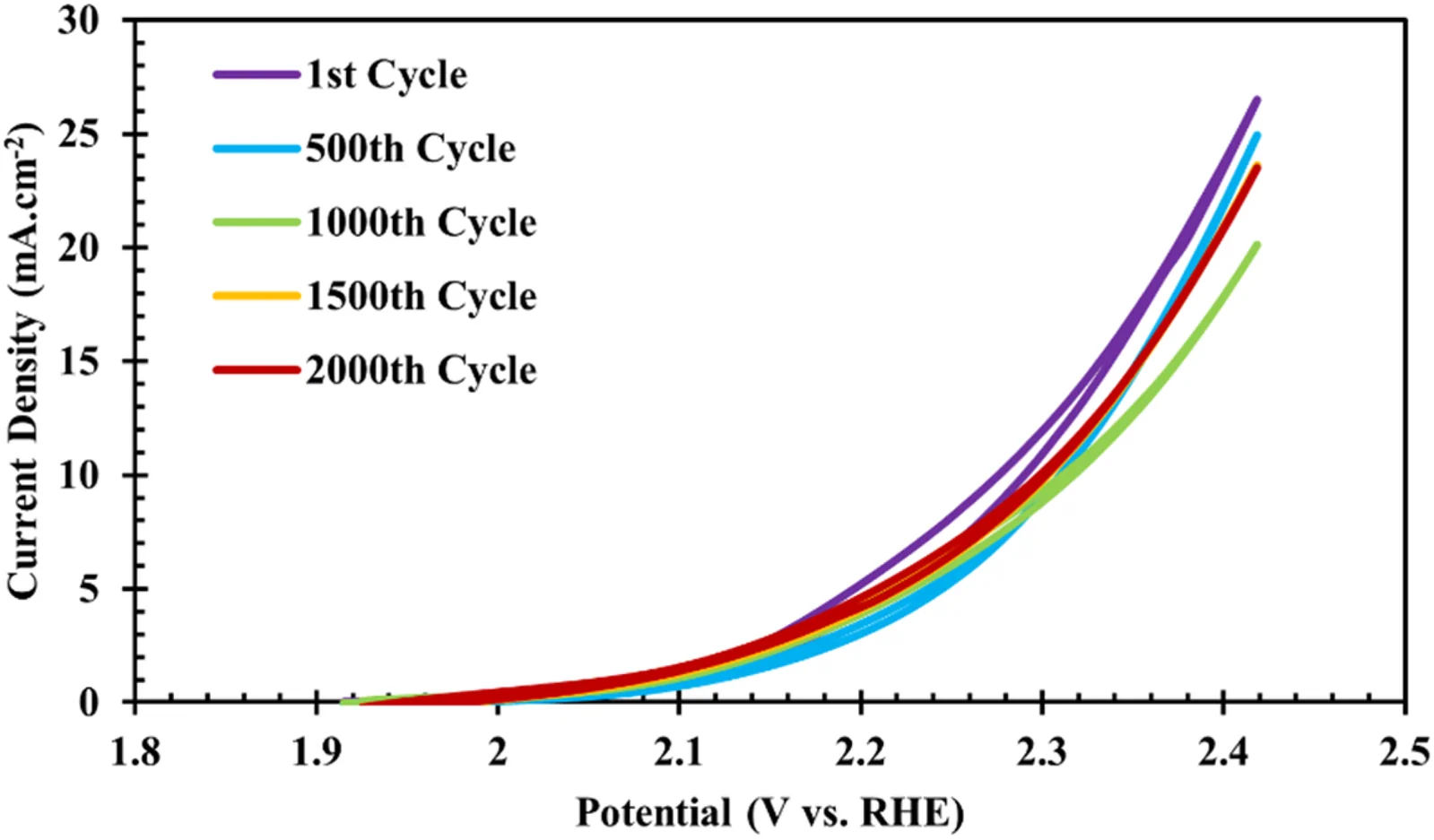
CV curves of the α + β-doped F & Fe anode in 0.5 M H_2_SO_4_ solution.

The initial potential drop observed in the CP test may be attributed to enhanced catalytic activity or electrolyte infiltration into surface porosity. To probe this phenomenon, 2000-cycle CV tests were conducted on the optimal α + β-doped F and Fe sample (Fig. 8). The electrode's activity decreased by 24% from the 1st to the 1000th cycle but increased by 17% from the 1000th to the 2000th cycle. This recovery in activity during the later stages corroborates the initial potential drop seen in the CP test. The overall results confirm the significant durability of the α + β-doped F and Fe anode in a 0.5 M H_2_SO_4_ environment over 2000 cycles.

**Fig. 9:**
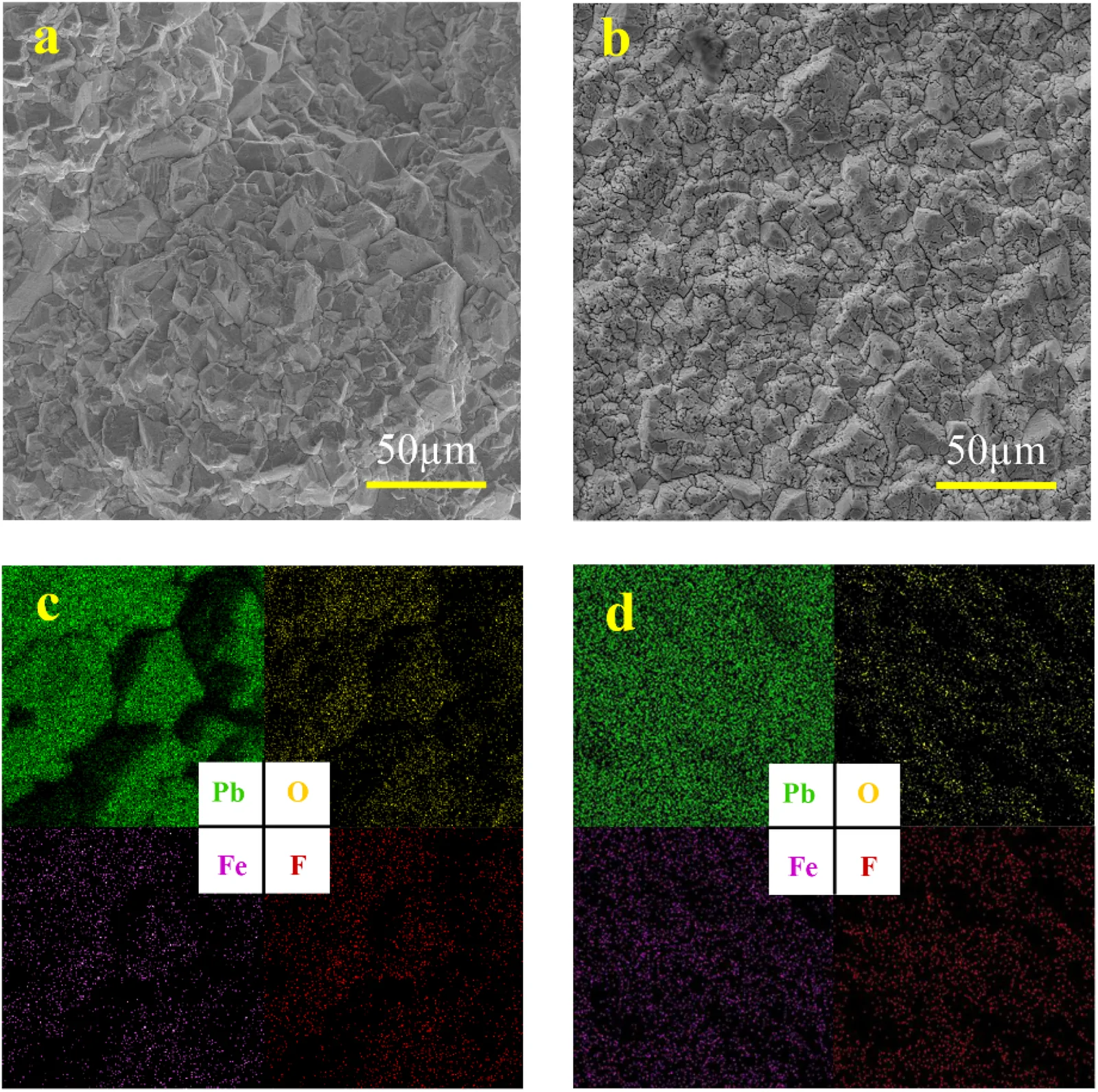
Surface morphology and corresponding EDS elemental mapping distributions of the optimized α + β-doped F and Fe electrode: FE-SEM micrographs in the (a) pristine state and (b) after the 2000-cycle durability test, along with the corresponding surface elemental mapping (Pb, O, Fe, and F) of the sample (c) before and (d) after the 2000-cycle test.

To examine structural changes and surface composition, FE-SEM analysis coupled with EDS elemental mapping was performed after the 2000-cycle durability test. Fig. 9 displays the surface morphology and elemental distributions. Fig. 9a and b show the topographic micrographs, while Fig. 9c and d illustrate the elemental mapping (Pb, O, Fe, and F) of the α + β-doped F and Fe sample before and after the 2000-cycle test, respectively. The micrographs reveal that the surface morphology remained highly stable after the 2000-cycle test, successfully preserving the typical pyramidal crystal structure of the lead dioxide coating. Additionally, the EDS mapping demonstrates a uniform and homogeneous distribution of elements across the outer surface, confirming that the chemical composition is well maintained even under harsh, long-term gas-evolution conditions.

## 4 Conclusions

In this study, a high-performance bilayer α + β PbO_2_ coating was successfully fabricated on a Ti/ATO substrate *via* a two-step electrodeposition process. The baseline introduction of the α-PbO_2_ underlayer effectively extended the electrode service life from less than 15 hours to 20 hours. To optimize the catalytic centers, the synergistic effects of fluorine (F^−^) and transition metal co-doping on the active outer β-PbO_2_layer were systematically investigated.

While individual fluorine doping enhanced stability at the expense of kinetics, co-doping with iron and fluorine yielded a refined, highly textured pyramidal surface matrix. XPS analysis confirmed the predominant integration of Fe^3+^ and F species, revealing a multi-valent Pb^2+^ and Pb^4+^ environment rich in structural oxygen vacancies. Small-amplitude cyclic voltammetry (SACV) demonstrated that this structural modification substantially expanded the electrochemical active surface area (ECSA). This expansion correlated directly with a drastic drop in charge transfer resistance (*R*_ct_) to just 20 Ω cm^2^, compared to 108 Ω cm^2^ for the undoped baseline.

Consequently, the optimized α + β-doped F and Fe anode achieved an outstanding accelerated service life of 66 hours at an intense current density of 3 A cm^−2^ a 3.3-fold increase over the undoped system. Furthermore, post-mortem FE-SEM and EDS mapping after a rigorous 2000-cycle voltammetric stress test confirmed the excellent retention of both the crystalline morphology and elemental distribution. These findings demonstrate that the synergistically co-doped F and Fe PbO_2_ anode is a highly active, robust, and promising alternative electrode material for the oxygen evolution reaction (OER) in aggressive acidic media.

## Conflicts of interest

There are no conflicts to declare.

## Supplementary Material

RA-016-D6RA05492G-s001

## Data Availability

All related data can be available upon request. Supplementary information (SI) is available. See DOI: https://doi.org/10.1039/d6ra05492g.
