# Supplementary material for: Enhancing the catalytic stability of lead dioxide electrodes for oxygen evolution reaction through α-PbO2 lining and β-PbO2 coating doped with F and Fe
Source: RSC Adv. 2026 Aug 10;16(40):46340–9. doi: 10.1039/d6ra05492g (PMC13455113; doi:10.1039/d6ra05492g)
Supplement: Supplementary file 1 [file RA-016-D6RA05492G-s001.pdf]

Supplementary Information (SI) for RSC Advances.
This journal is © The Royal Society of Chemistry 2026

Supporting Information

Enhancing the catalytic stability of lead dioxide electrodes for oxygen evolution reaction through α-PbO_2 lining and β-PbO_2 coating Doped with F and Fe

Mohanna Amini, Milad Rezaei*

Department of Materials and Metallurgical Engineering, Amirkabir University of Technology (Tehran Polytechnic), Hafez Ave., P.O. Box 15875-4413, Tehran, Iran

***Corresponding author:** M. Rezaei (miladrezaei@aut.ac.ir)

Fig. S1 presents the high-magnification FE-SEM micrographs (scale bar: 2 μm) of the fabricated coatings. A direct comparison between Figs. S1a and S1b reveals a distinct grain-refinement effect, where the crystalline features become noticeably finer upon the individual incorporation of fluorine into the lead dioxide matrix. Furthermore, these micrographs demonstrate that the optimized α + β-doped F and Fe sample exhibits a highly textured surface characterized by enhanced nanoscale roughness and a more irregular pyramidal morphology compared to the other dopant configurations. This modified surface architecture contributes to a larger electrochemical active surface area, which is highly beneficial for accelerating the oxygen evolution reaction (OER) kinetics.

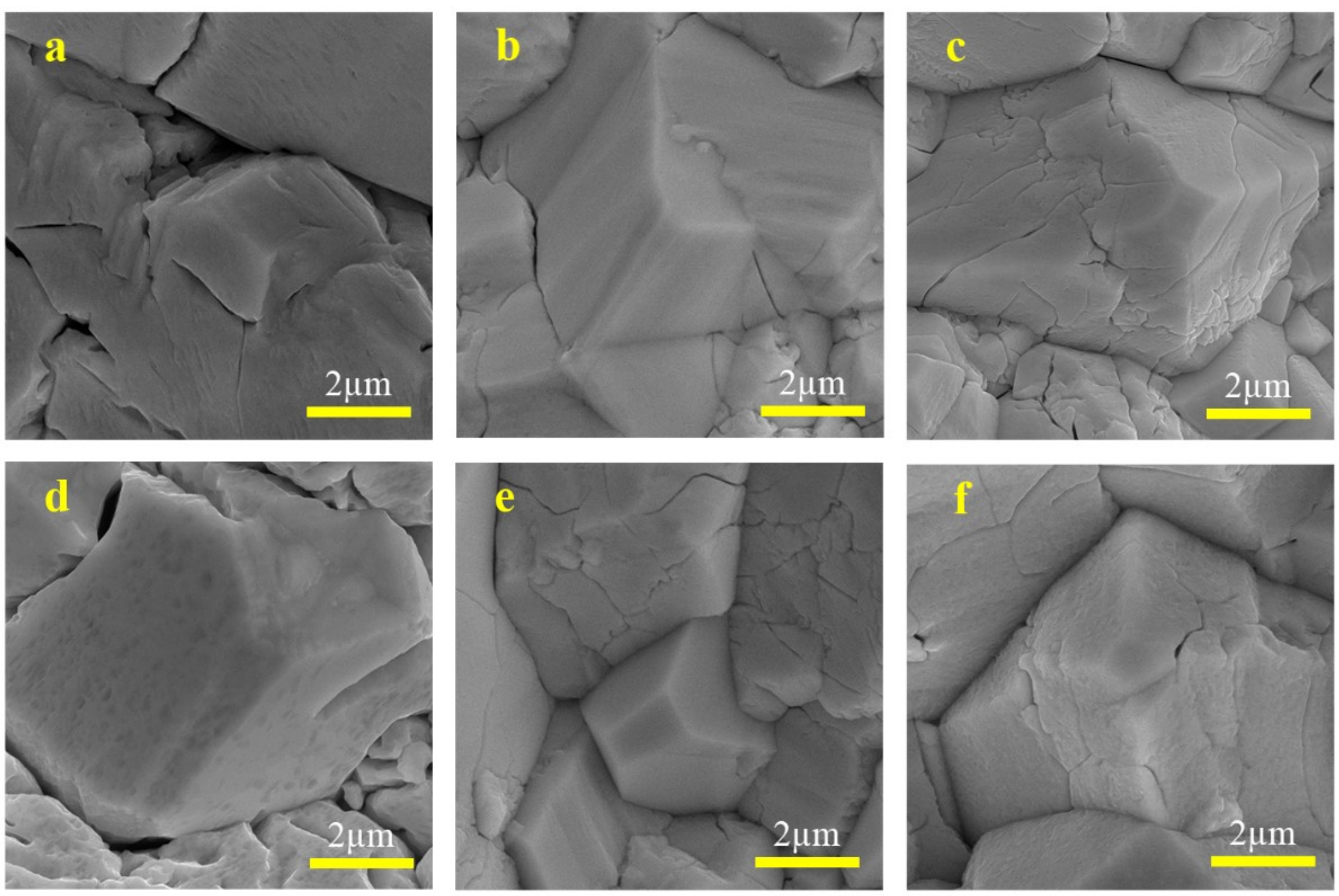

Fig. S1. FE-SEM micrographs (scale bar: 2 μm) for the samples, respectively; (a) α + β, (b) α + β-doped F, (c) α + β-doped F and Cu, (d) α + β-doped F and Ni, (e) α + β-doped F and Co, and (f) α + β-doped F and Fe.

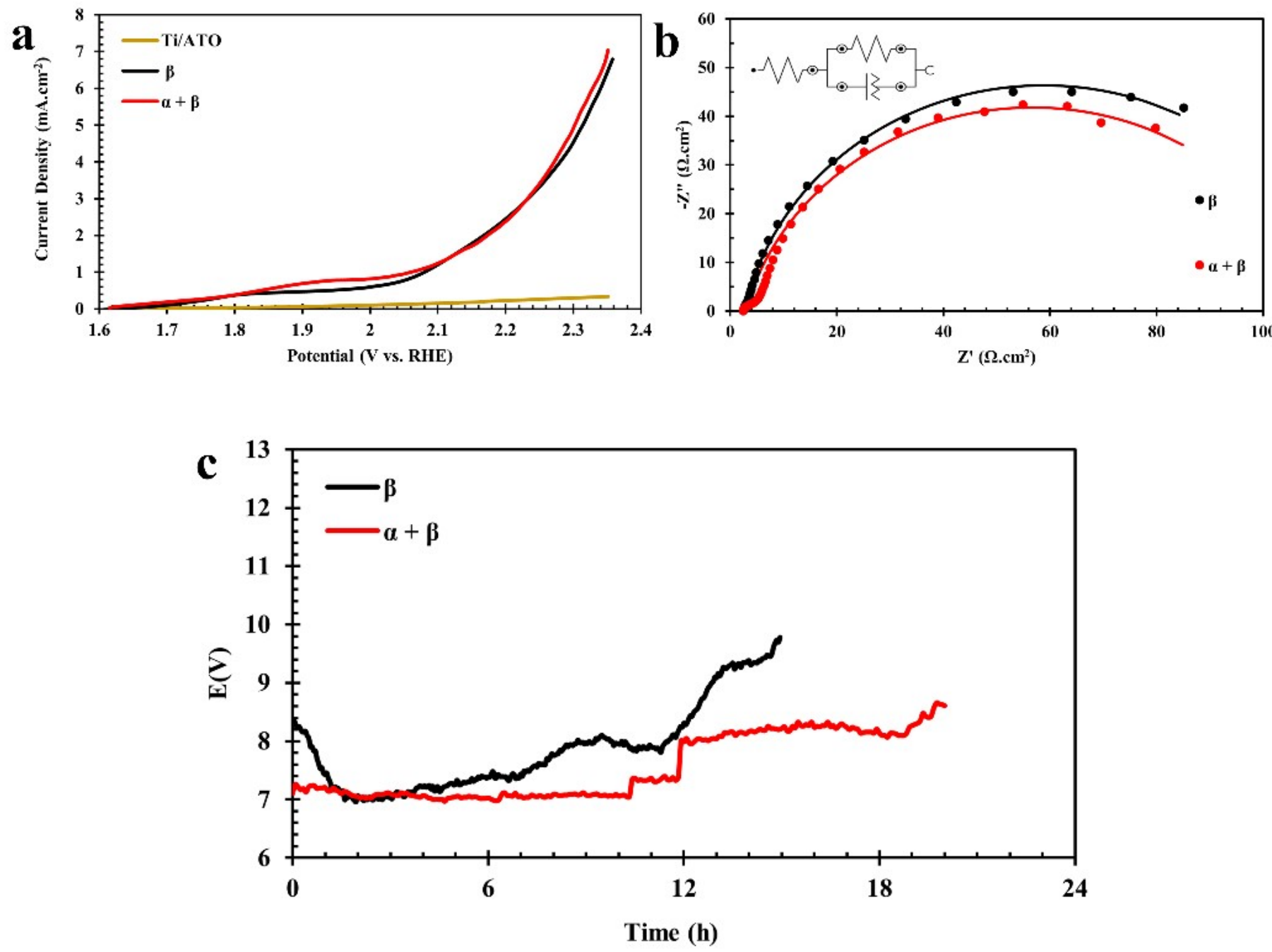

Fig. S2. Comparative electrochemical evaluation of the single-layer β-PbO_2 and bilayer α + β electrodes through (a) LSV curves, (b) EIS Nyquist plots, and (c) chronopotentiometry (CP) accelerated stability profiles.

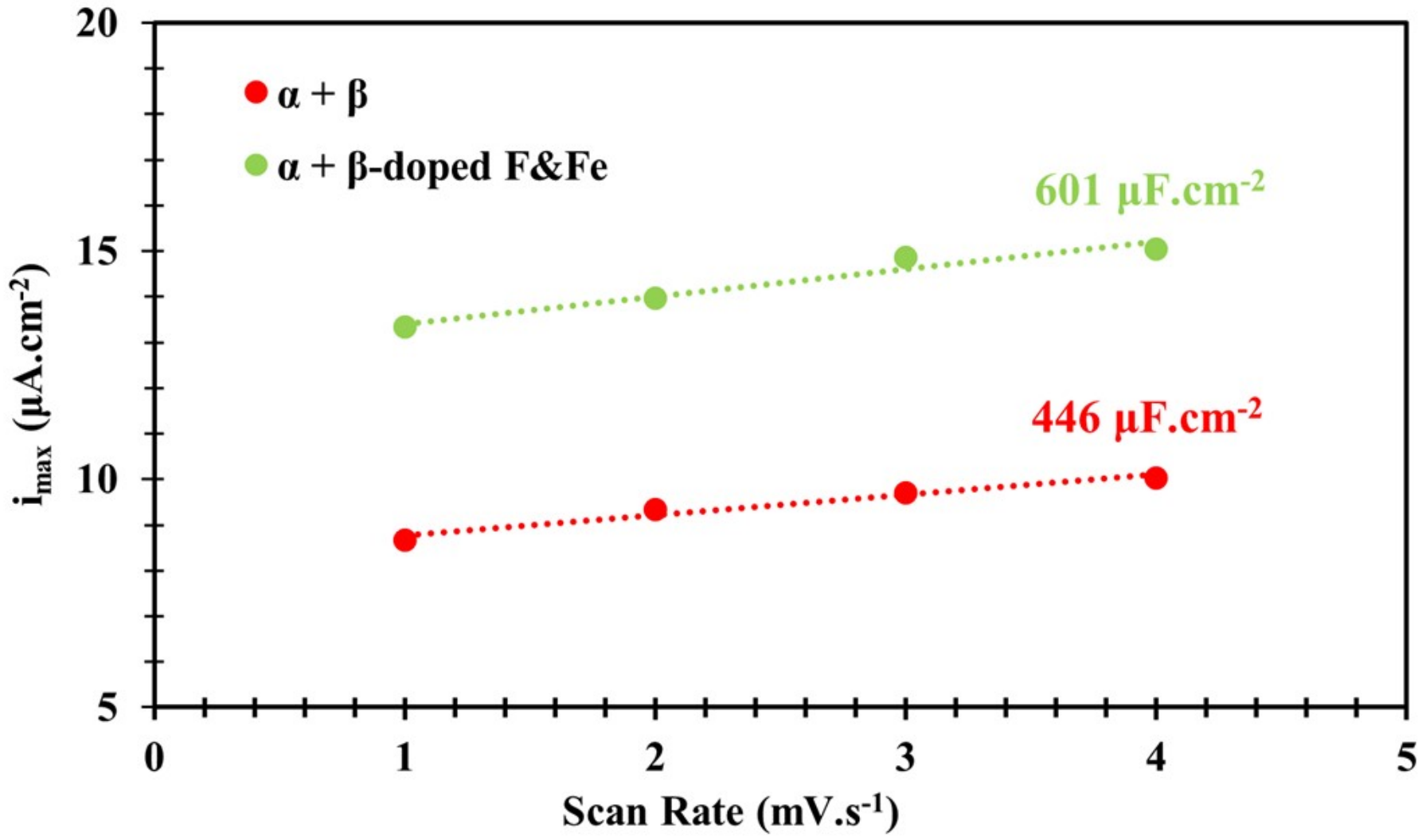

Fig. S3. Double-layer capacitance (C_{dl}) estimation for the α+ β and α + β-doped F and Fe electrodes, obtained by plotting the capacitive current density (i_{max}) as a function of the voltammetric scan rate extracted from small-amplitude cyclic voltammetry (SACV) measurements.
